# Supplementary material for: Untrackable distal ejecta on planetary surfaces
Source: Nat Commun. 2023 Mar 1;14:1173. doi: 10.1038/s41467-023-36771-y (PMC9977847; doi:10.1038/s41467-023-36771-y)
Supplement: Supplementary file 1 — Supplementary Information [file 41467_2023_36771_MOESM1_ESM.pdf]

Supplementary Materials for

Untrackable distal ejecta on planetary surfaces

Rui Xu[1†] and Zhiyong Xiao[1,2†*], Fanglu Luo[1], Yichen Wang[1], Jun Cui[1,2]

[1] Planetary Environmental and Astrobiological Research Laboratory, School of Atmospheric Sciences, Sun Yat-sen University, Zhuhai, China.

[2] CAS Center for Excellence in Comparative Planetology, Hefei, China.

[†] These authors contributed equally: Rui Xu and Zhiyong Xiao.

[*] **Corresponding author**: Zhiyong Xiao (xiaozhiyong@mail.sysu.edu.cn)

This PDF file includes:
Supplementary Figs.1–8
Supplementary Table 1

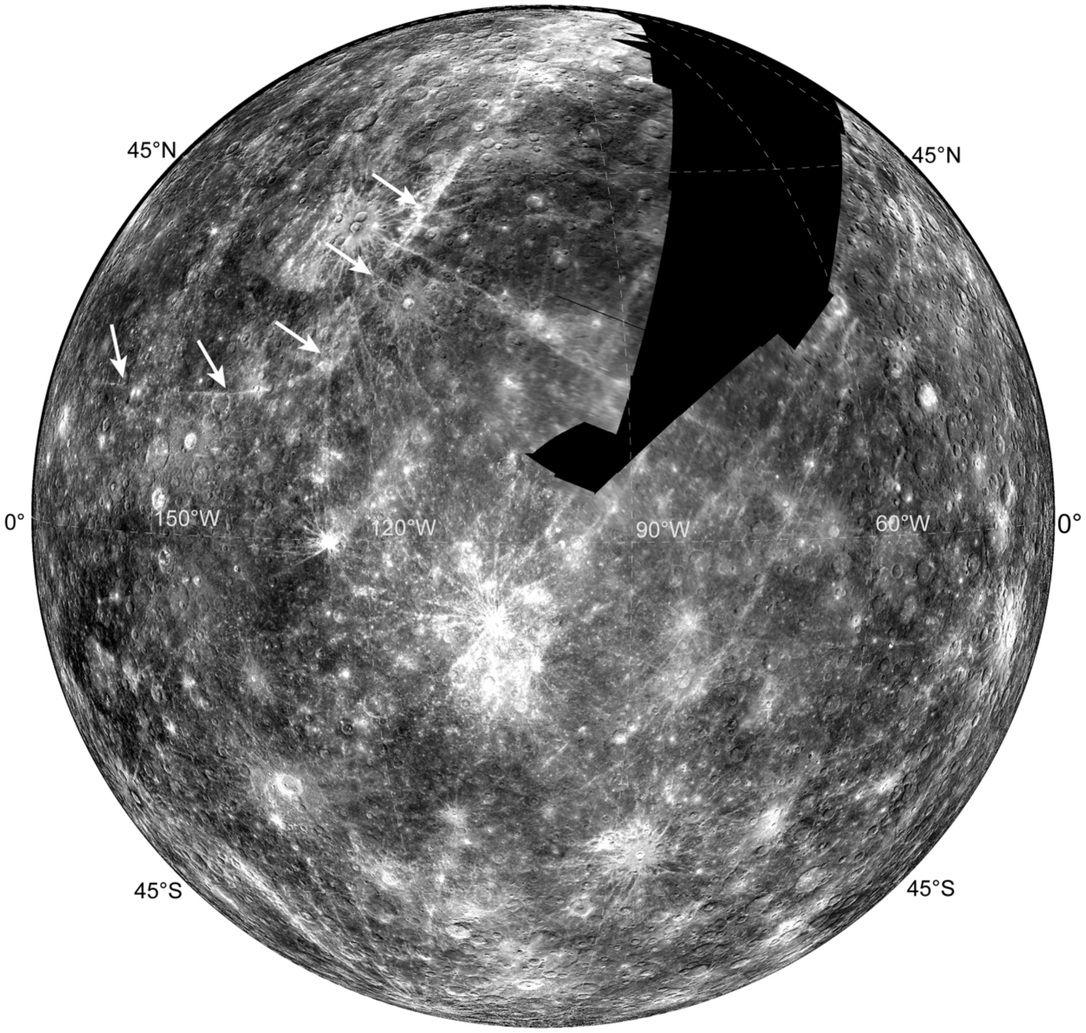

Supplementary Fig. 1 | The hyperbola-shaped ray (white arrows) on Mercury as revealed by the Mariner 10 spacecraft. This mosaic contains all images of Mercury returned by Mariner 10. Image IDs used for this figure are listed in Supplementary Table 1.

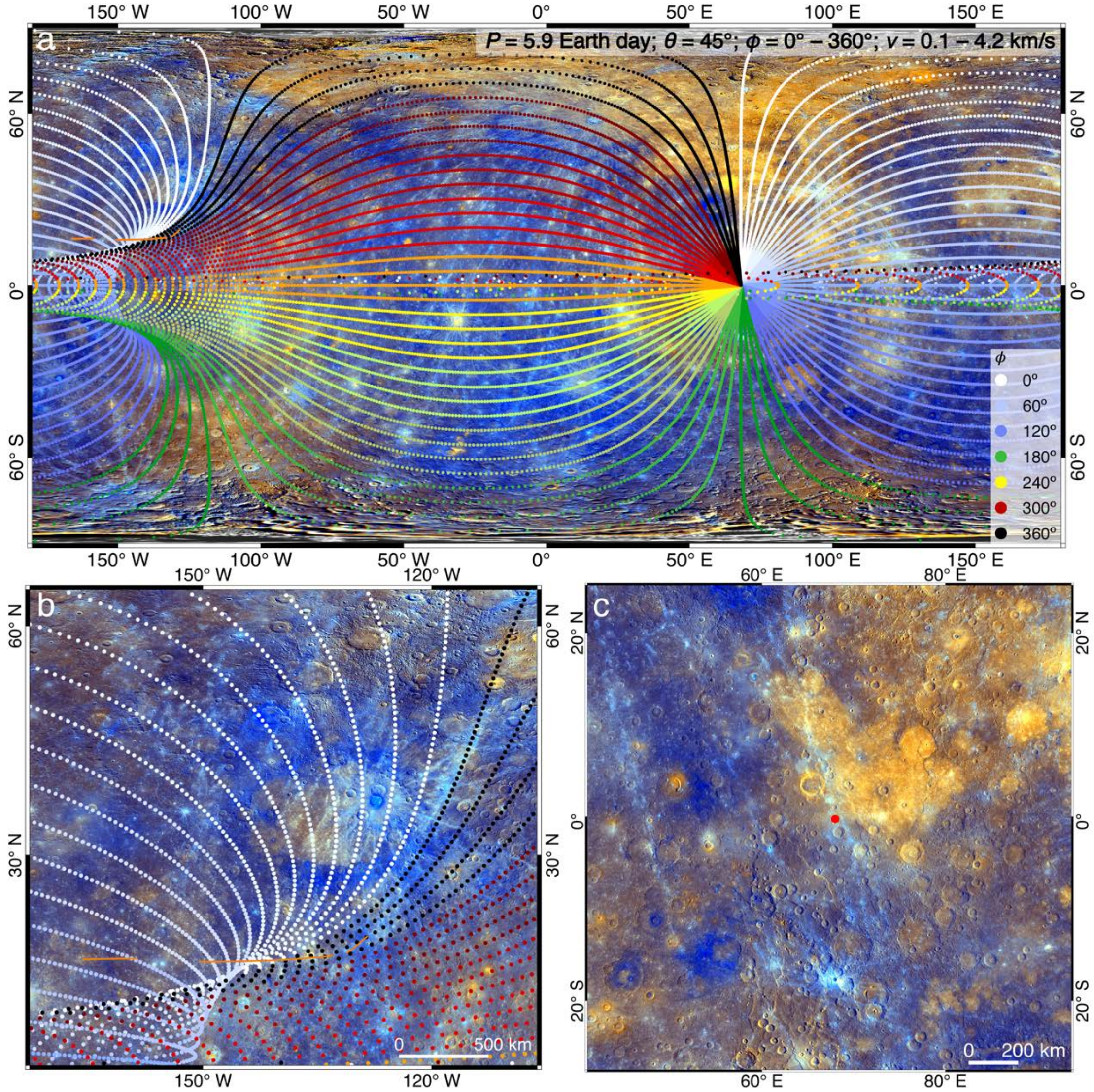

Supplementary Fig. 2 | The curved ray was not formed by a potential source impact at low latitudes of the eastern hemisphere. **a** Landing positions of ejecta excavated by the hypothesized impact on a faster rotating Mercury. The curved ray (brown lines) was interpreted to be formed by distal ejecta from this hypothesized source impact[1]. The modelled ejecta have an assumed angle of ejection (θ) of 45°, and the rotation period of Mercury is assumed to be 5.9 Earth days[1]. Ejecta are colored based on their azimuths of excavation (ϕ). **b** Enlarged view for landing positions of ejecta at this curved ray. **c** No potential source craters with impact rays is visible at the earlier predicted region of impact that may form this curved ray[1]. Image IDs used for this figure are listed in Supplementary Table 1.

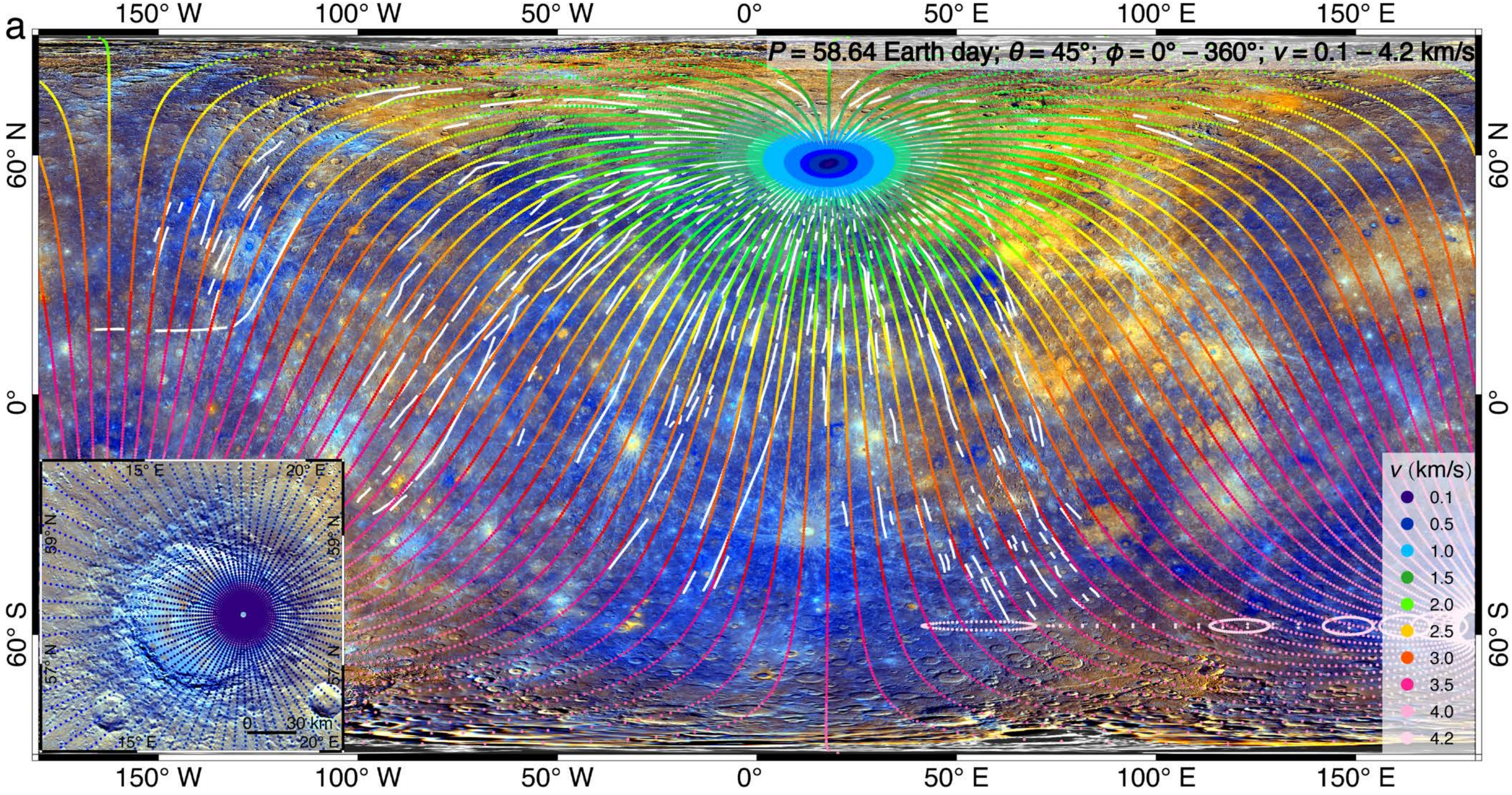
a
150° W
100° W
50° W
0°
50° E
100° E
150° E
60° N
0°
60° S
P = 58.64 Earth day; θ = 45°; ϕ = 0° – 360°; v = 0.1 – 4.2 km/s
v (km/s)
0.1
0.5
1.0
1.5
2.0
2.5
3.0
3.5
4.0
4.2
15° E
20° E
59° N
57° N
0
30 km

b

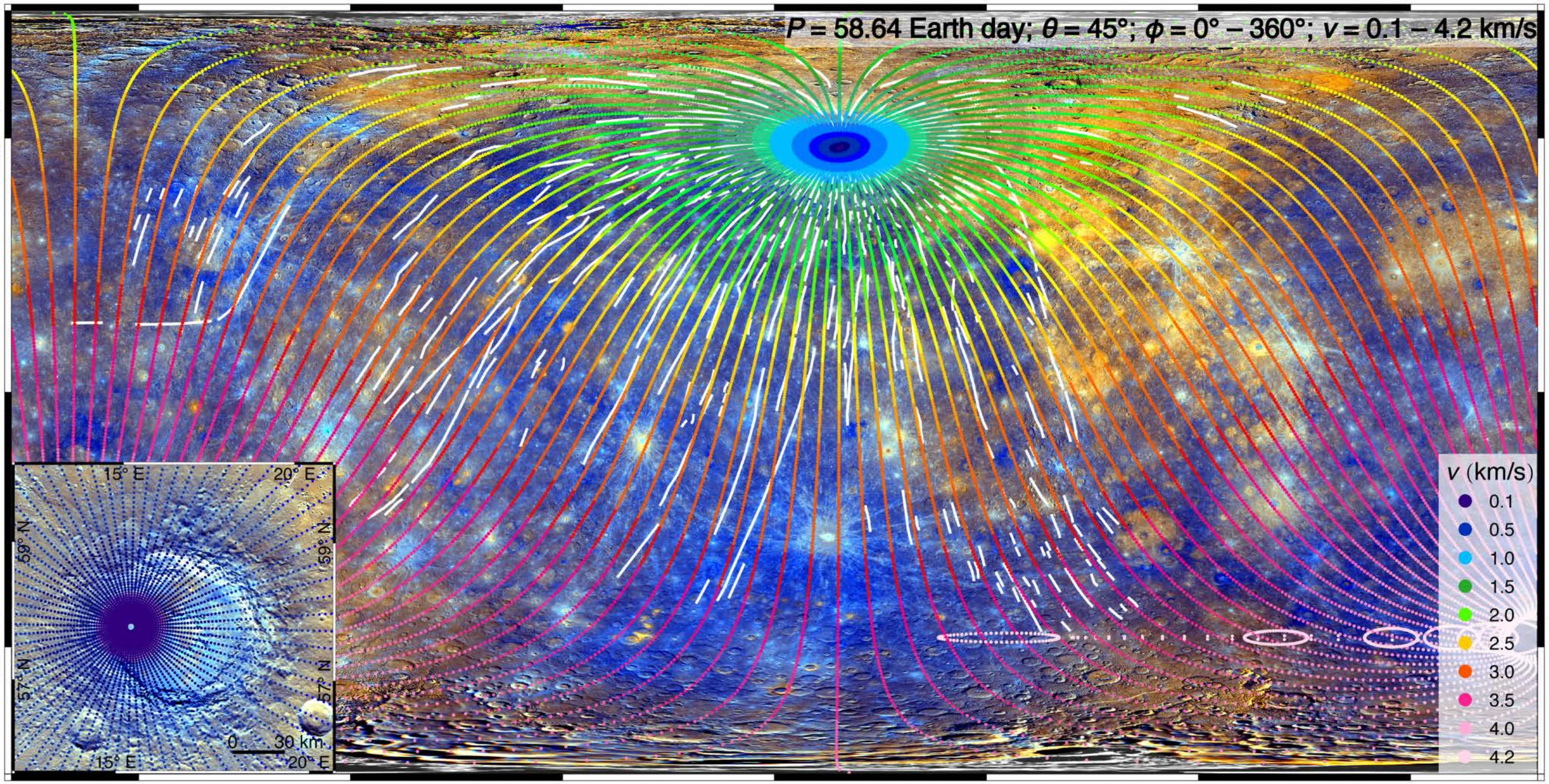

150° W
100° W
50° W
0°
50° E
100° E
150° E
P = 58.64 Earth day; θ = 45°; ϕ = 0° – 360°; v = 0.1 – 4.2 km/s
60° N
0°
60° S
15° E
20° E
59° N
57° N
0
30 km
v (km/s)
0.1
0.5
1.0
1.5
2.0
2.5
3.0
3.5
4.0
4.2

c

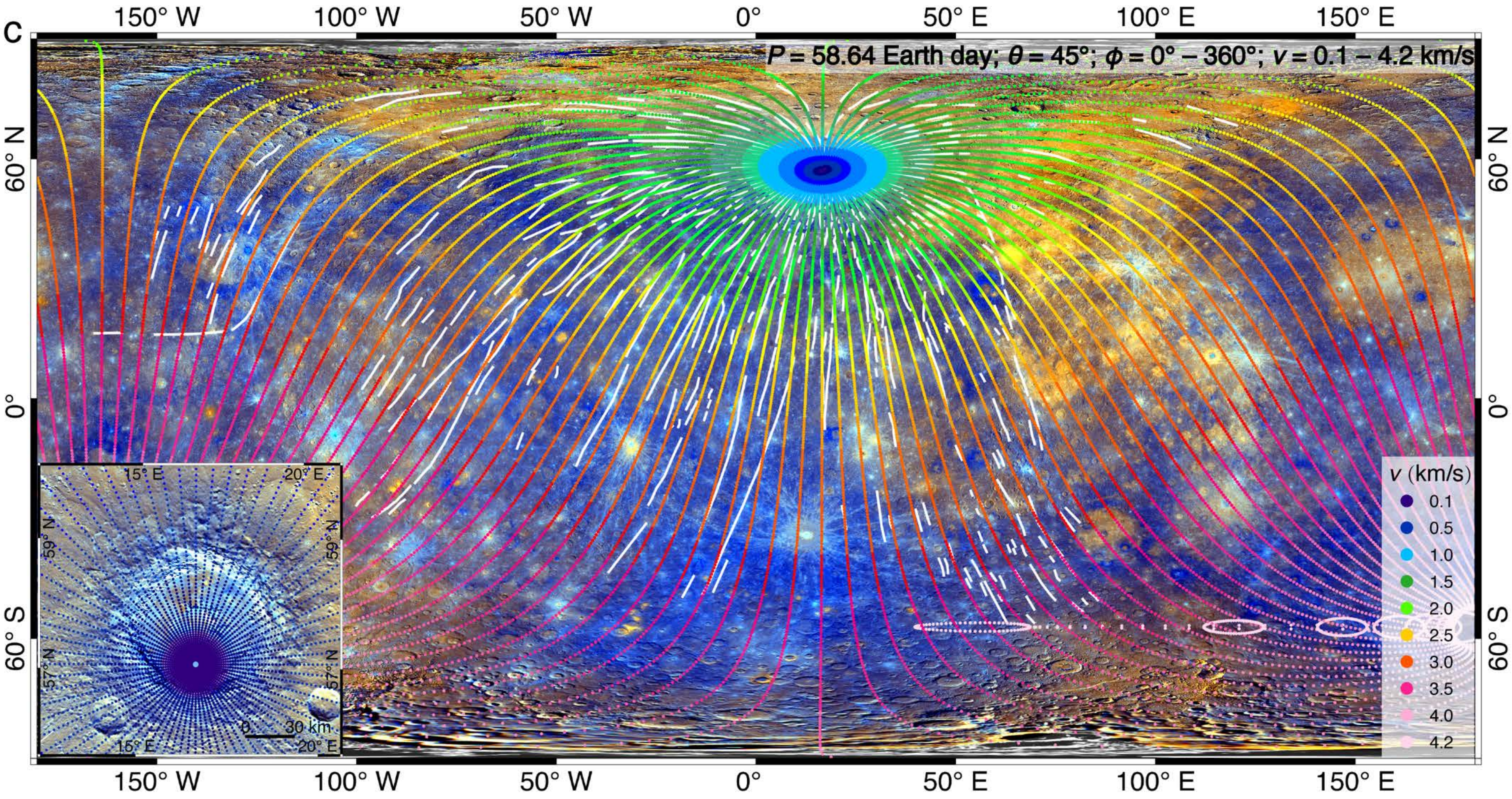

150° W
100° W
50° W
0°
50° E
100° E
150° E
P = 58.64 Earth day; θ = 45°; ϕ = 0° – 360°; v = 0.1 – 4.2 km/s
60° N
0°
60° S
15° E
20° E
59° N
57° N
0
30 km
v (km/s)
0.1
0.5
1.0
1.5
2.0
2.5
3.0
3.5
4.0
4.2

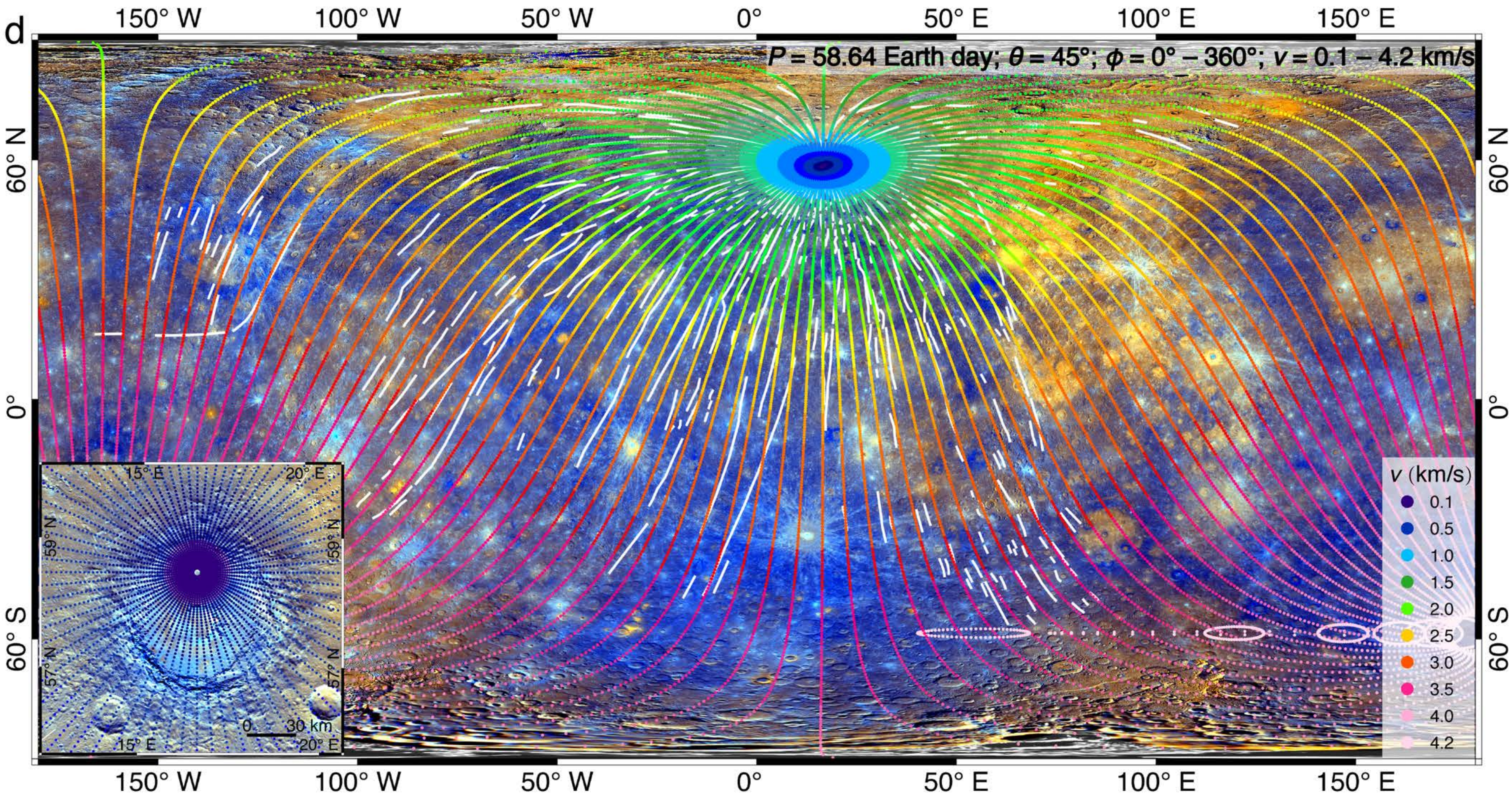

d
P = 58.64 Earth day; θ = 45°; ϕ = 0° – 360°; v = 0.1 – 4.2 km/s
150° W
100° W
50° W
0°
50° E
100° E
150° E
60° N
0°
60° S
v (km/s)
0.1
0.5
1.0
1.5
2.0
2.5
3.0
3.5
4.0
4.2
15° E
20° E
59° N
57° N
0
30 km

Supplementary Fig. 3 | Landing positions of ejecta that were excavated from different locations in the transient cavity of the Hokusai crater. a–d Cases with assumed positions of excavation from the eastern, western, southern and northern rims of the transient crater of Hokusai (see Methods), respectively. A uniform ejection angle of θ =45° is used in the modelling, and the rotation period of Mercury (*P*) is the same with it is today. The ejecta particles are colored based on their ejection velocities (*v*). Regardless of the different launch positions, most visible rays of Hokusai (white lines) are consistent with spatial patterns of landing positions of the modelled ejecta. Image IDs used for this figure are listed in Supplementary Table 1.

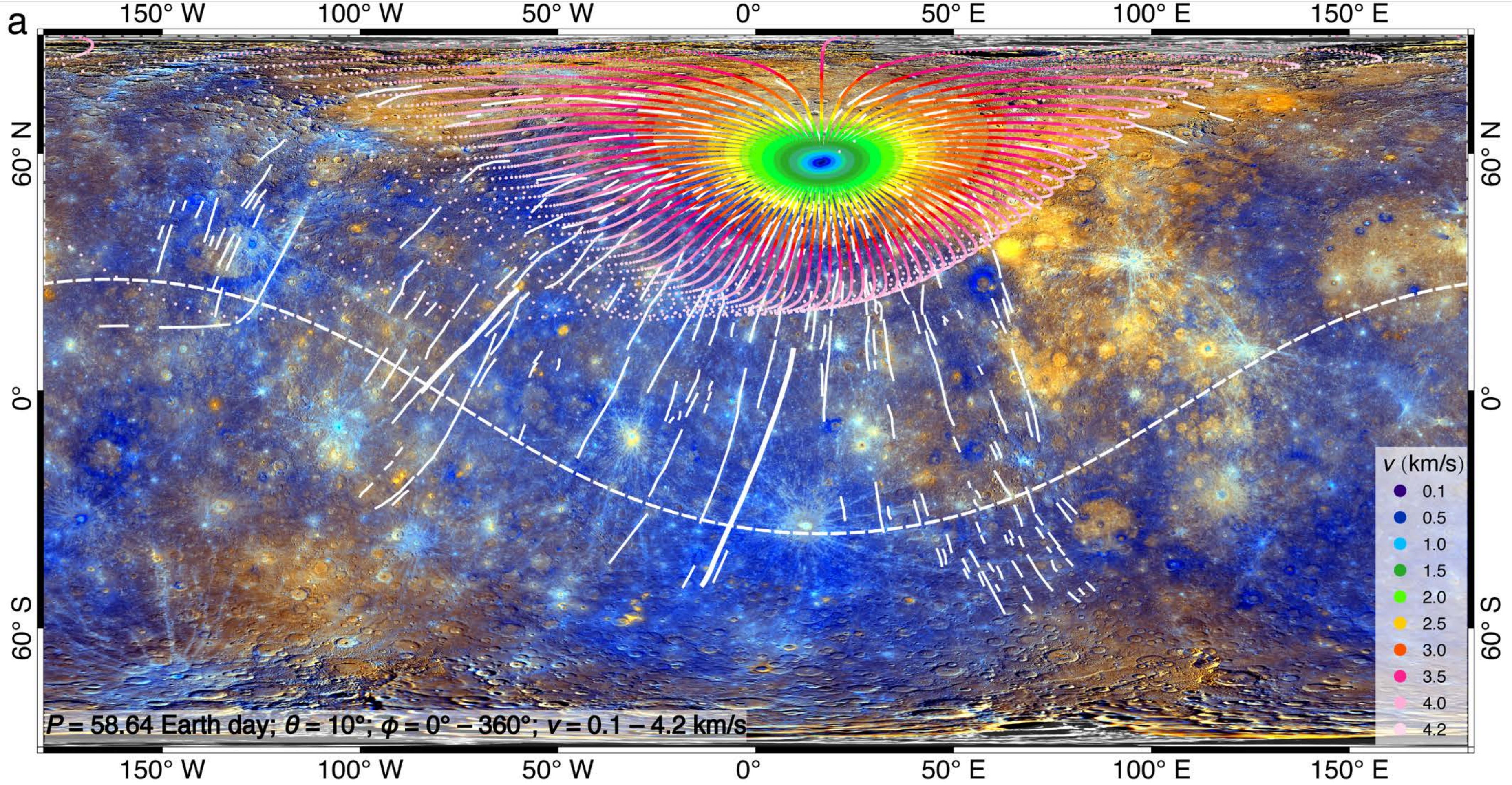

a
150° W
100° W
50° W
0°
50° E
100° E
150° E
60° N
0°
60° S
v (km/s)
0.1
0.5
1.0
1.5
2.0
2.5
3.0
3.5
4.0
4.2
P = 58.64 Earth day; θ = 10°; ϕ = 0° – 360°; v = 0.1 – 4.2 km/s

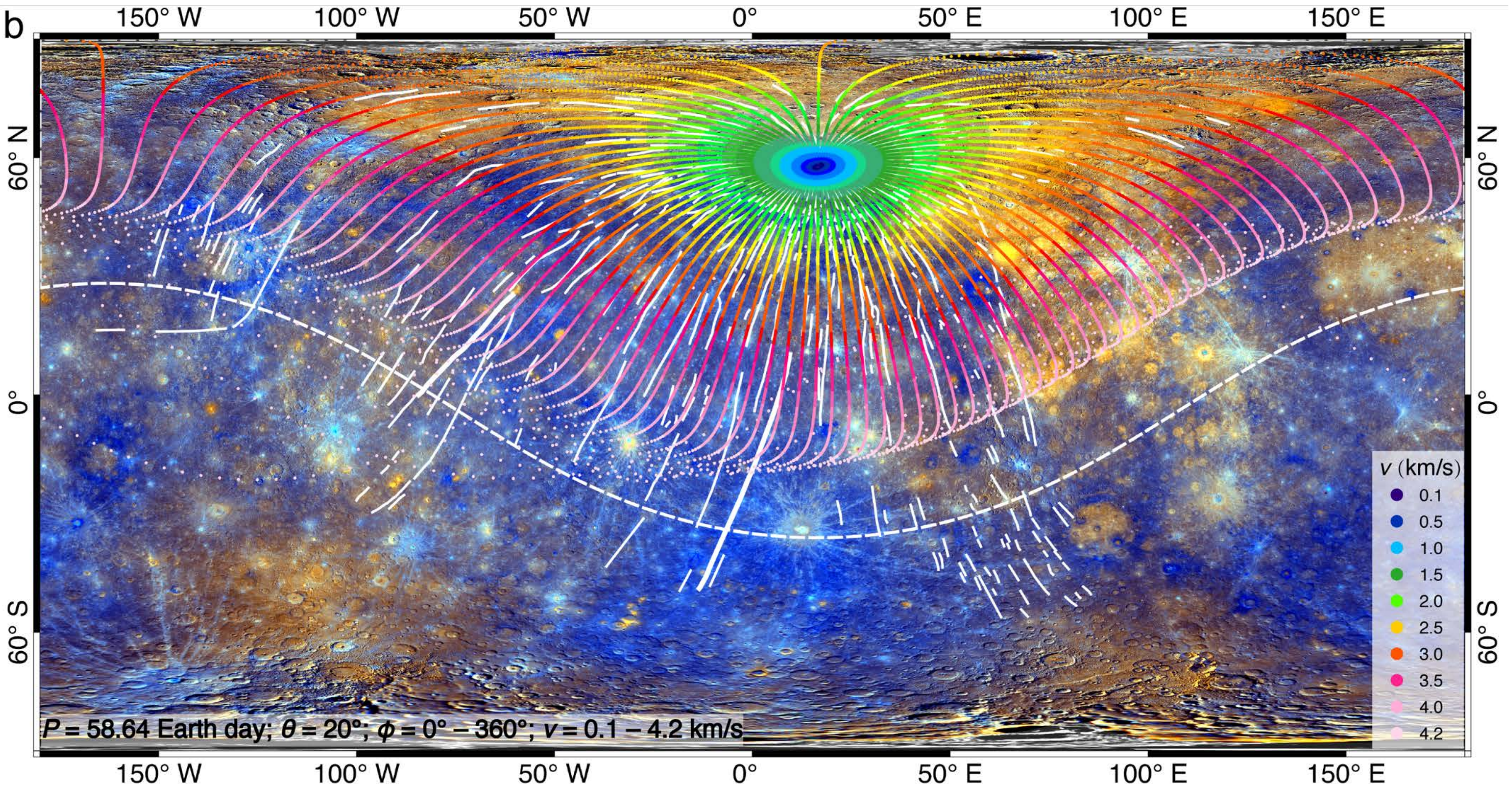
b
150° W
100° W
50° W
0°
50° E
100° E
150° E
60° N
0°
60° S
P = 58.64 Earth day; θ = 20°; φ = 0° − 360°; v = 0.1 − 4.2 km/s
v (km/s)
0.1
0.5
1.0
1.5
2.0
2.5
3.0
3.5
4.0
4.2

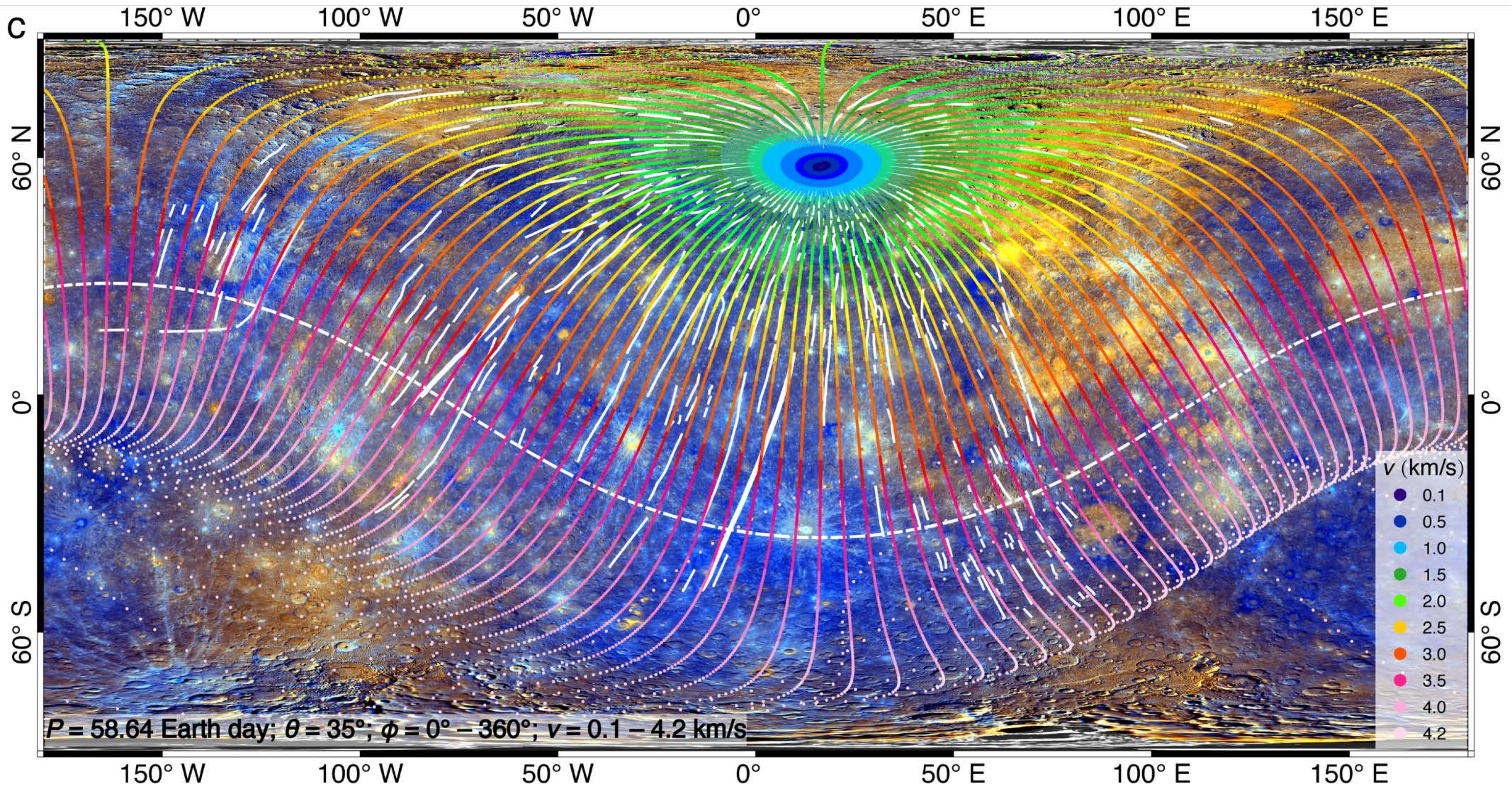

c
150° W
100° W
50° W
0°
50° E
100° E
150° E
60° N
0°
60° S
P = 58.64 Earth day; θ = 35°; ϕ = 0° − 360°; v = 0.1 − 4.2 km/s
v (km/s)
0.1
0.5
1.0
1.5
2.0
2.5
3.0
3.5
4.0
4.2

d

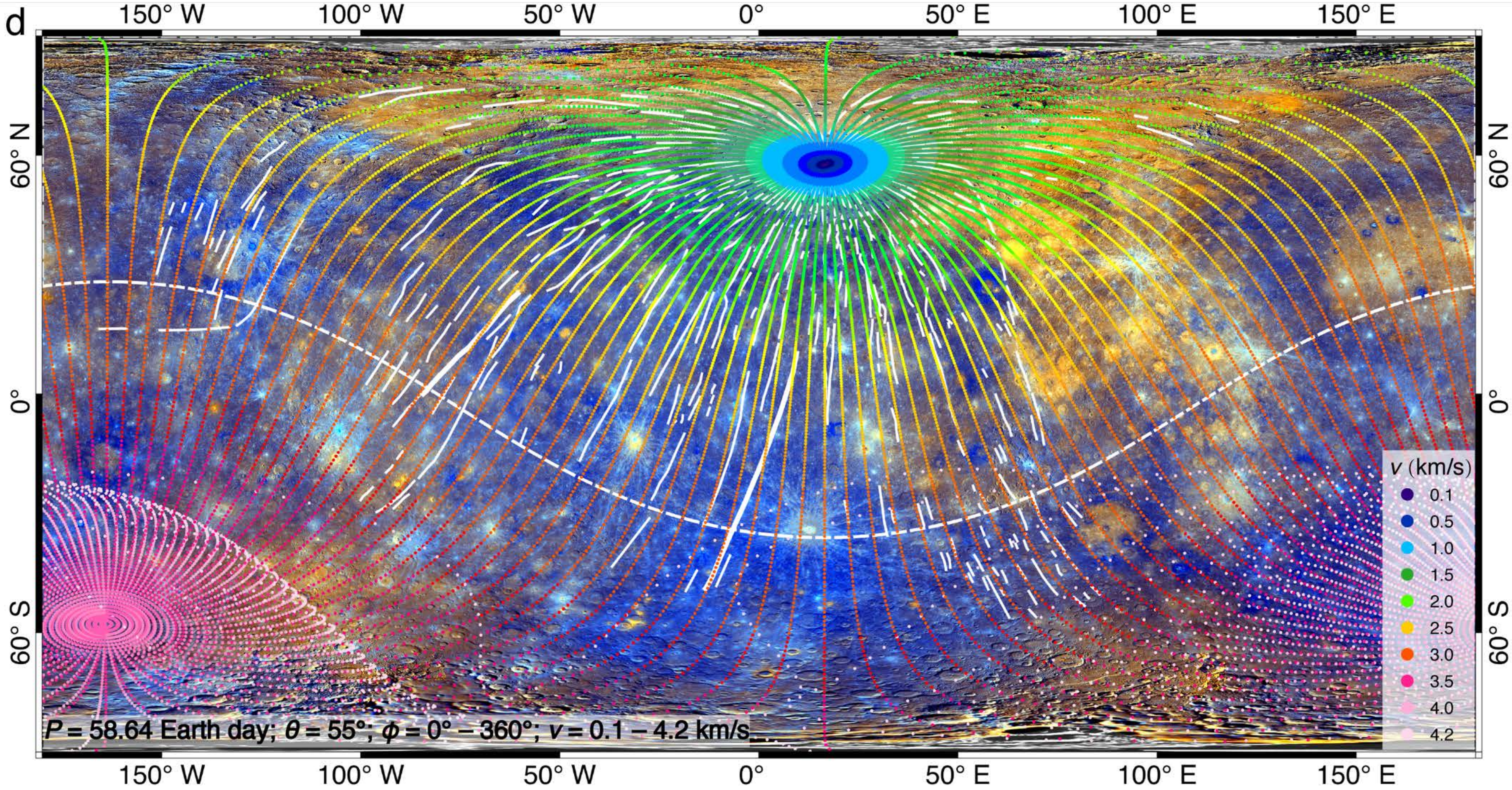
150° W
100° W
50° W
0°
50° E
100° E
150° E
60° N
0°
60° S
v (km/s)
0.1
0.5
1.0
1.5
2.0
2.5
3.0
3.5
4.0
4.2
P = 58.64 Earth day; θ = 55°; φ = 0° – 360°; v = 0.1 – 4.2 km/s

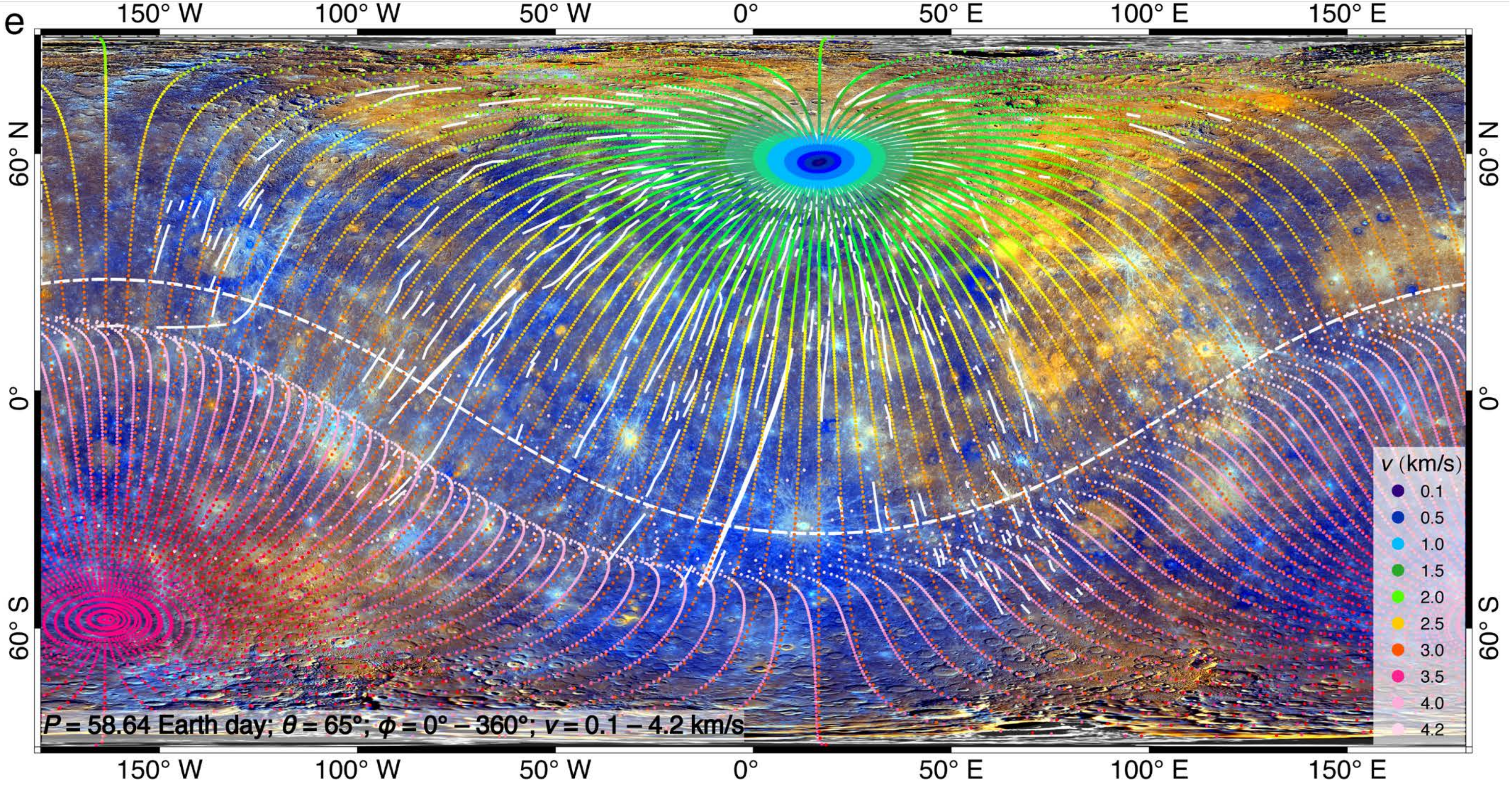
e
150° W
100° W
50° W
0°
50° E
100° E
150° E
60° N
0°
60° S
v (km/s)
0.1
0.5
1.0
1.5
2.0
2.5
3.0
3.5
4.0
4.2
P = 58.64 Earth day; θ = 65°; ϕ = 0° – 360°; v = 0.1 – 4.2 km/s

Supplementary Fig. 4 | Landing positions of Hokusai's ejecta that have different assumed ejection angles. a–e Cases with assumed ejection angles (θ) of 10°, 20°, 35°, 55°, and 65°, respectively. The ejecta particles were assumed to be excavated from the center of Hokusai, and the landing positions are colored based on their ejection velocities (v). Most visible rays of Hokusai (white solid lines) that are less than 4000 km away from Hokusai (white dashed curve) are consistent with spatial patterns of landing positions of ejecta that have ejection angles of 35° to 55° (**c–d**). Image IDs used for this figure are listed in Supplementary Table 1.

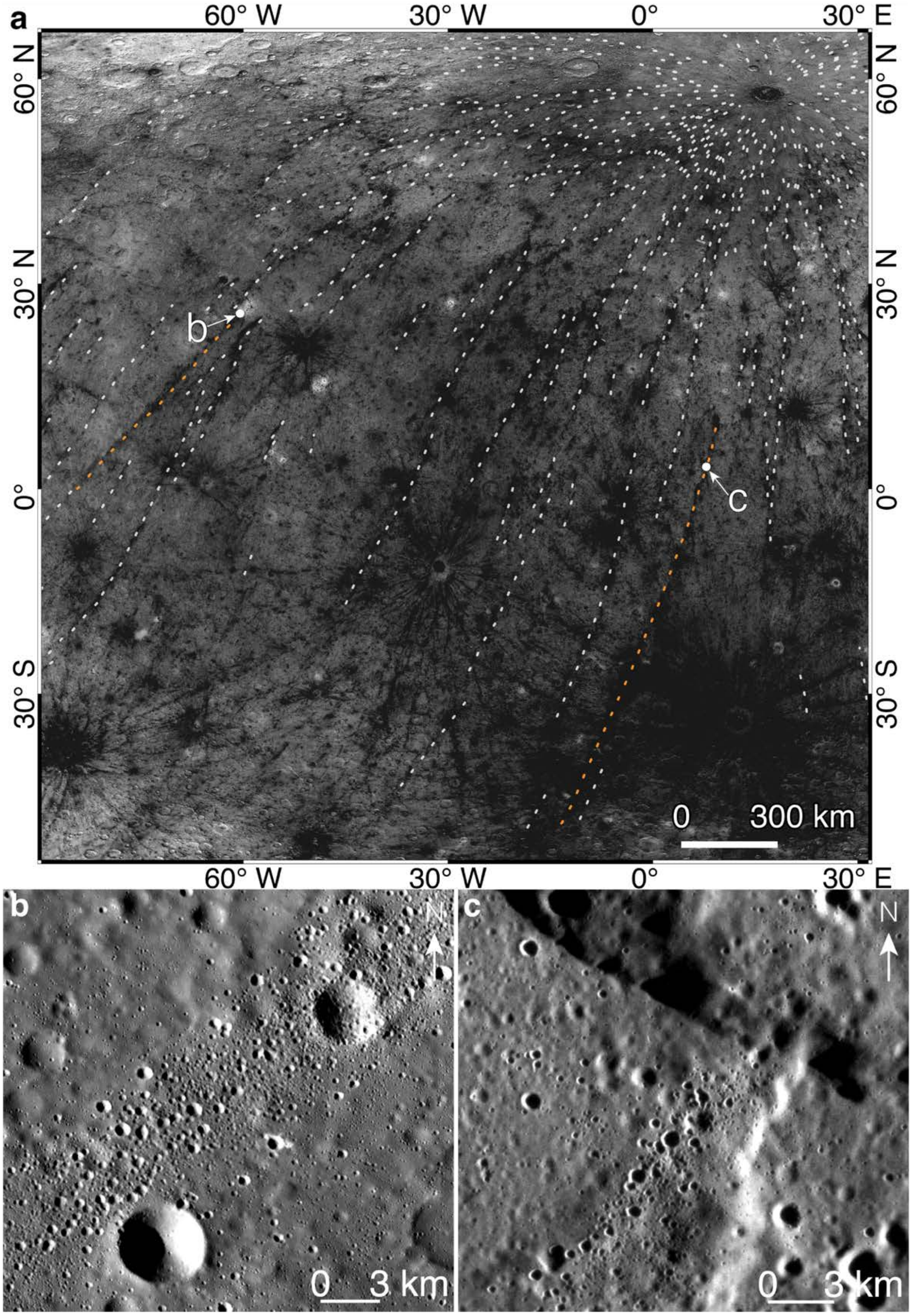

Supplementary Fig. 5 | Examples of other non-radial rays of the Hokusai crater that were formed by ejecta with abnormal ejection angles. a Two distal rays exhibit non-radial orientations with respect to the Hokusai crater (brown dashed lines). Their intersection points with adjacent radial rays (white dashed lines) are far from the Hokusai crater. **b, c** Morphology of secondaries in these rays. The chains of secondaries have the same orientation with the local ray segments, and the preferential depositional direction of ejecta that were formed by these secondaries also indicates the same azimuth of landing. Image IDs used for this figure are listed in Supplementary Table 1.

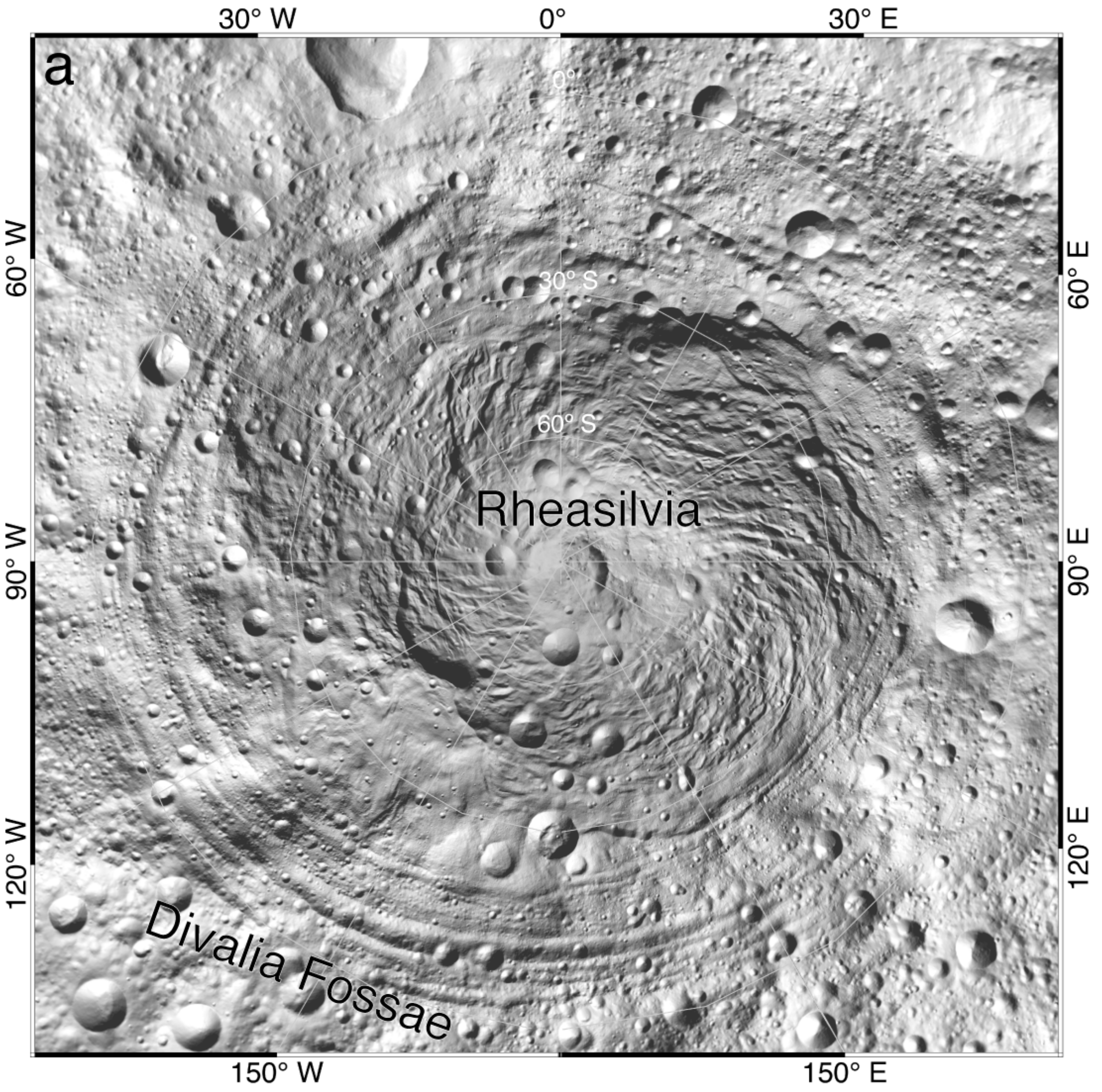
30° W
0°
30° E
a
0°
60° W
60° E
30° S
60° S
Rheasilvia
90° W
90° E
120° W
120° E
Divalia Fossae
150° W
150° E

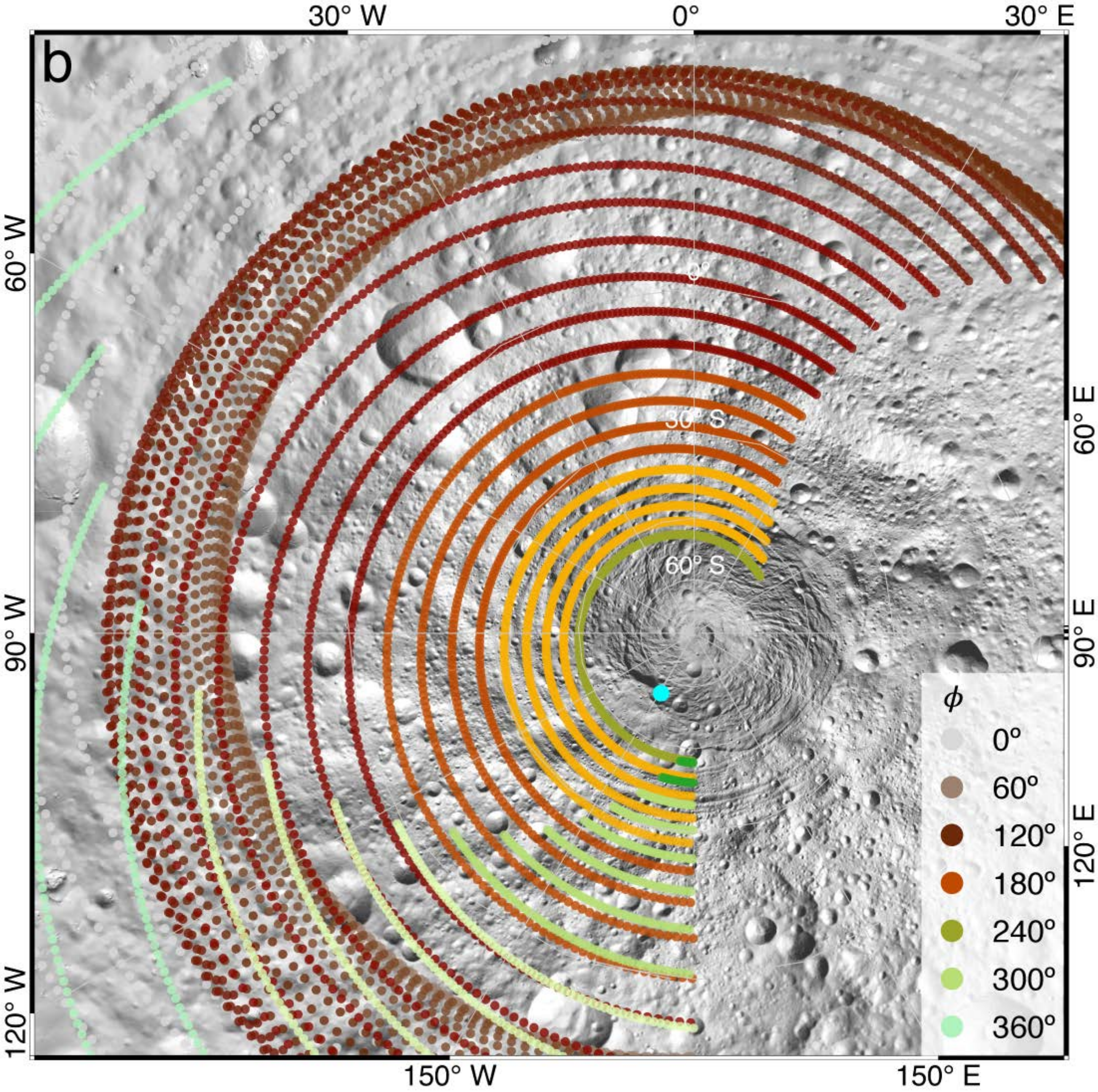
b
30° W
0°
30° E
60° W
90° W
120° W
150° W
150° E
60° E
90° E
120° E
0°
30° S
60° S
ϕ
0º
60º
120º
180º
240º
300º
360º

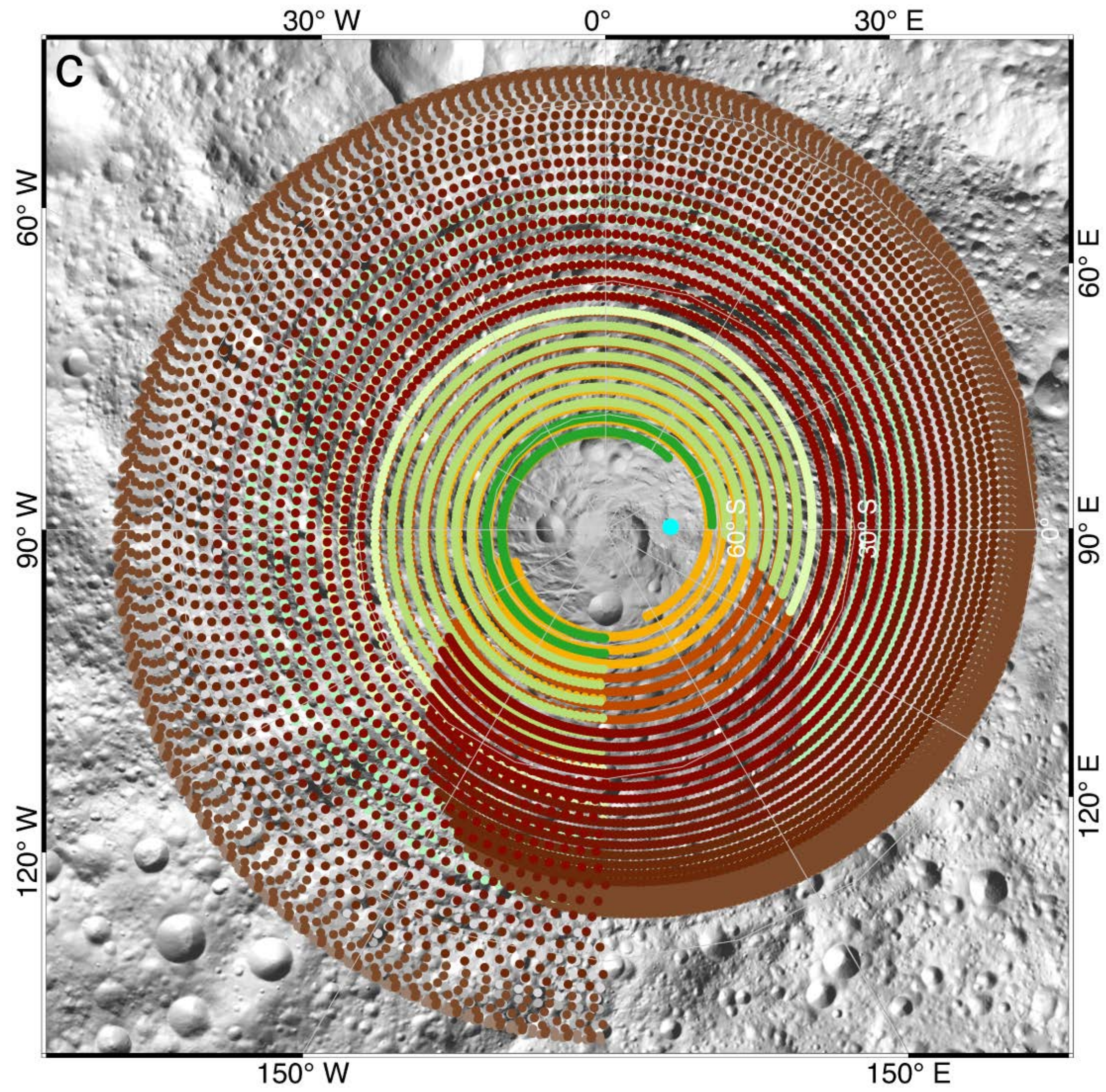

Supplementary Fig. 6 | A secondary impact origin for some of the circumferential troughs around the walls and rims of the Rheasilvia basin. a The morphology of the troughs that include the Divalia Fossae. **b, c** Landing positions for simulated ejecta of the Rheasilvia basin that have ejection angles of θ=45° and θ=20°, respectively. In both cases, only ejecta that have flight times larger than 5 Earth hours are shown, as the formation of the Rheasilvia basin lasted about 5 Earth hours[2]. Besides, particles with landing positions in the interior of Rheasilvia (south of 65° S) are not shown in the plot. Cyan dots are the assumed positions of ejection. Ejecta excavated from a same azimuth of ejection were coded using a same color (the two panels share the same color legend). Image IDs used for this figure are listed in Supplementary Table 1.

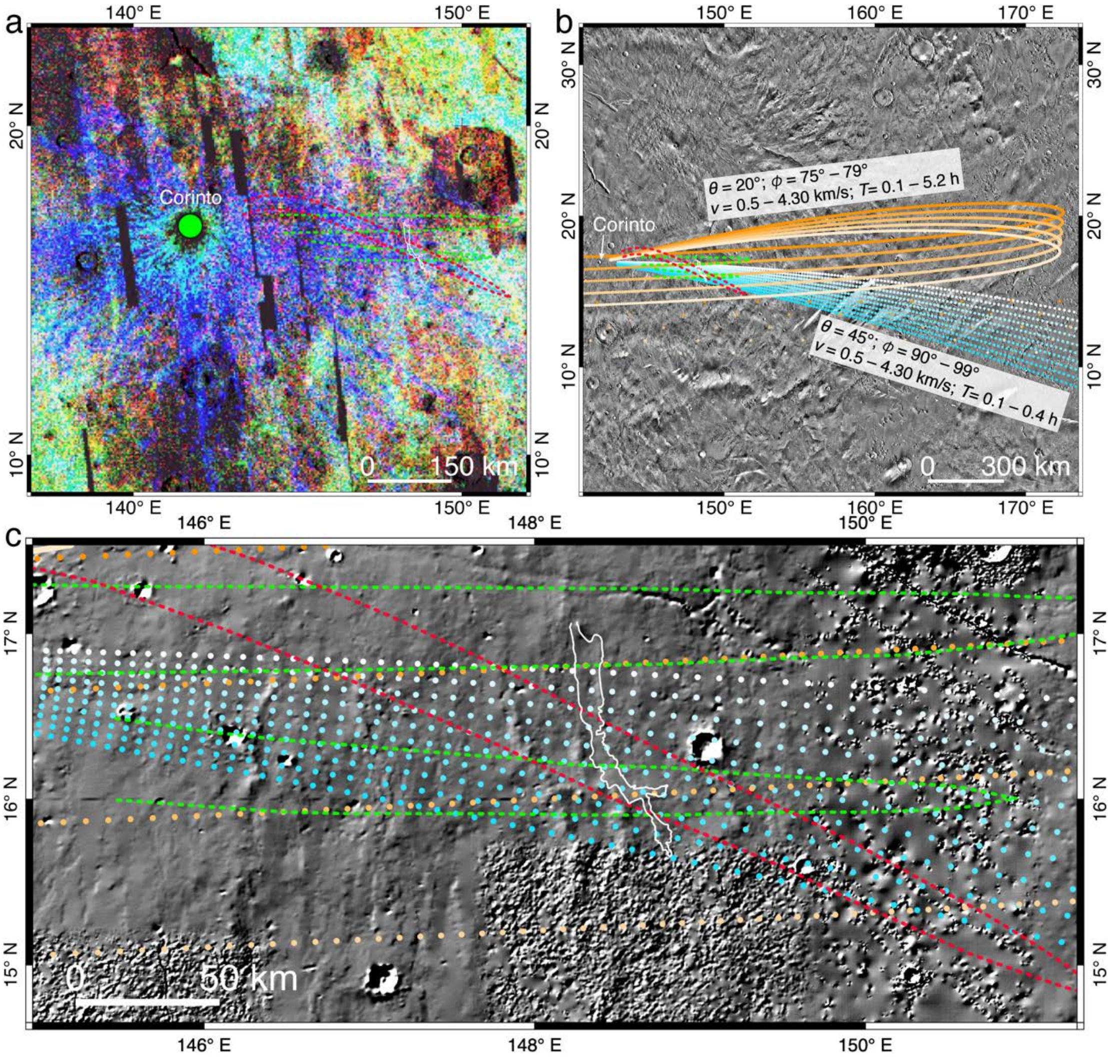

Supplementary Fig. 7 | Overlapping of rays of distal ejecta formed by a same crater on Mars. a Detected rays of secondaries formed by the Corinto crater on Mars. The different color of this figure denotes spatial densities of impact craters that have different diameter ranges[3]. The green dashed curves mark two examples of rays of secondaries that are more-or-less radial to the Corinto crater. The red dashed curve marks a curved ray of secondaries. **b, c** Possible ejection conditions for ejecta that formed the marked non-radial and radial rays of the Corinto crater. This model does not include the effect of the Mars atmosphere on the ballistic trajectory of ejecta. Ejecta with different excavation conditions (i.e., annotations) may land at the same location but from different azimuths (the two panels share the same color legend). Seen in high-resolution images (Fig. 10c), visible secondaries in the mapped rays are mostly circular in shapes and relatively isolated in the spatial distribution, so their azimuths of landing are not obvious as evident from the morphology. Therefore, the simulations here do not intent to untangle the best-fit ejection conditions for each secondary crater in the radial or non-radial rays. If additional observational constraint(s) would resolve the ejection conditions of the secondaries (e.g., v, θ and ϕ), the model is capable to better reproduce the ballistic trajectory. Image IDs used for this figure are listed in Supplementary Table 1.

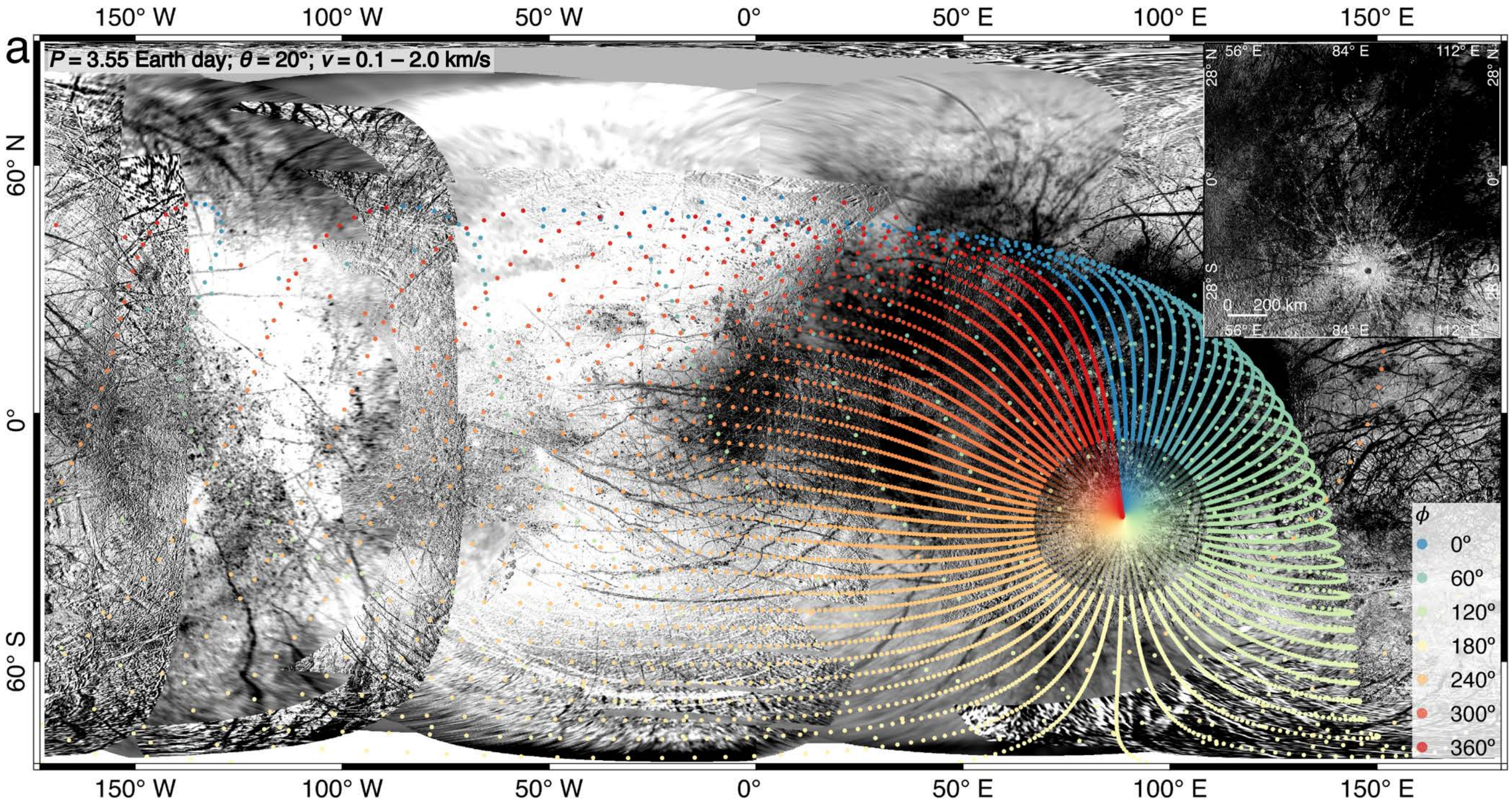
a
P = 3.55 Earth day; θ = 20°; v = 0.1 – 2.0 km/s
150° W
100° W
50° W
0°
50° E
100° E
150° E
60° N
0°
60° S
56° E
84° E
112° E
28° N
28° S
0
200 km
ϕ
0°
60°
120°
180°
240°
300°
360°

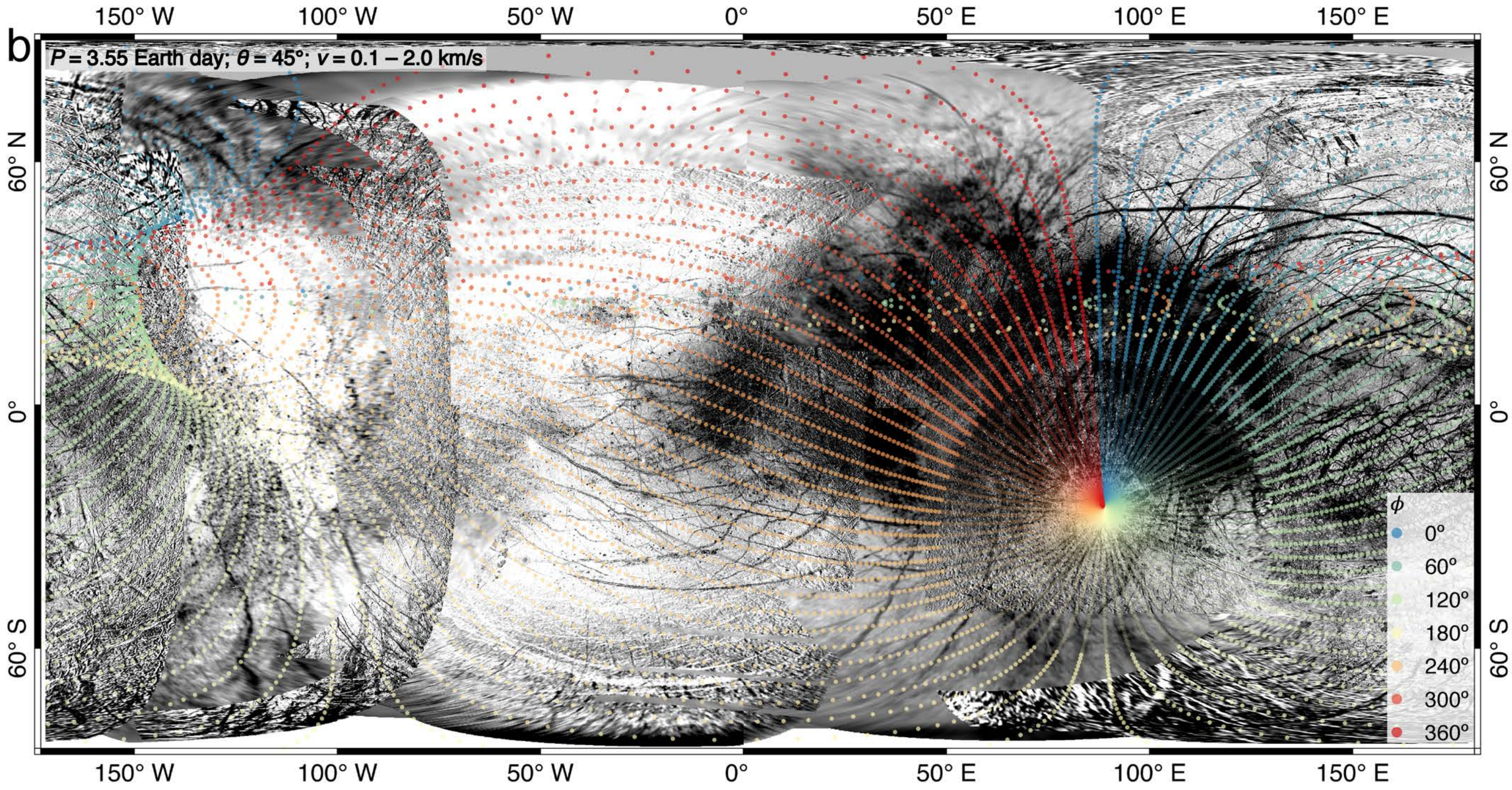

Supplementary Fig. 8 | Landing positions of distal ejecta formed by the Pywll crater on Jupiter's icy moon Europa. **a, b** Cases for ejecta with assumed ejection angles of 20° and 45°, respectively. The other parameters used in the model are annotated. The inset figure in (**a)** shows the radial distributions of rays of the Pywll crater. Europa has a highly dynamic and young ice crust[4], and most craters less than 1 km in diameters were believed

to be secondaries formed by the rayed crater Pwyll[5]. Due to the fast rotational period of Europa, landing positions of ejecta formed by the Pywll crater are substantially deflected from radial distributions, and some of them may land at the same location from different azimuths, especially for ejecta with steep ejection angles. Therefore, it is predictable that spatial densities of small impact craters on Europa might not reflect actual ages of the underlying terranes. Image IDs used for this figure are listed in Supplementary Table 1.

Supplementary Table 1 l IDs and available addresses of data used in this study. S means Supplementary.

Figure number	Data ID	Map projection	Pixel scale	Available address
**Fig. 1a, Fig. 4a, Fig. 5, S Fig. 2a, S Fig. 3, S Fig. 4**	MDIS BDR Global Mosaic	simple cylindrical	166 m/pixel	https://messenger.jhuapl.edu/Explore/Images.html#global-mosaics
**Fig. 1a,b, Fig. 6, S Fig. 2, S Fig. 3, S Fig. 4**	MDIS WAC Enhanced Color	simple cylindrical	665 m/pixel	https://astrogeology.usgs.gov/search/map/Mercury/Messenger/Global/Mercury_MESSENGER_MDIS_Basemap_EnhancedColor_Mosaic_Global_665m
**Fig. 1c**	MDIS Mercury Maturity Index	simple cylindrical	665 m/pixel	https://doi.org/10.6084/m9.figshare.20722891.v4
**Fig. 1d, Fig. 4, Fig. 5, S Fig. 5a**	global map of slopes of reflectance spectra at visible to near-infrared wavelengths	simple cylindrical	665 m/pixel	https://doi.org/10.6084/m9.figshare.20722891.v4
**Fig. 2a**	MDIS MD3Color Global Mosaic	simple cylindrical	665 m/pixel	https://messenger.jhuapl.edu/Explore/Images.html#global-mosaics
**Fig. 2b, Fig. 9a,b**	LROC WAC_EMP_3BAND_E300N3150_064P	Equirectangular	473 m/pixel	http://pds.lroc.asu.edu/data/LRO-L-LROC-5-RDR-V1.0/LROLRC_2001/EXTRAS/BROWSE/WAC_EMP/
**Fig. 2c**	Mars Odyssey THEMIS-IR Night 60N60S Mosaic	simple cylindrical	100 m/pixel	https://pds-imaging.jpl.nasa.gov/volumes/ody.html
**Fig. 2d**	MDIS NAC EN1014559767M	Equirectangular	30 m/pixel	https://pdsimage2.wr.usgs.gov/archive/mess-e_v_h-mdis-2-edr-rawdata-v1.0/
**Fig. 2e**	Selene Kaguya Terrain Camera TCO_MAPm04_N21E291N18E294SC	Mercator	7.4 m/pixel	https://darts.isas.jaxa.jp/planet/pdap/selene/index.html.en
**Fig. 2f**	MRO CTX D01_027556_1865_XN_06N194W	Equirectangular	5.7 m/pixel	https://pds-imaging.jpl.nasa.gov/portal/mro_mission.html
**Fig. 3a**	MDIS NAC EN0227683745M; EN0227683702M	Equirectangular	118 m/pixel	https://pdsimage2.wr.usgs.gov/archive/mess-e_v_h-mdis-2-edr-rawdata-v1.0/
**Fig. 3b**	MDIS NAC	Equirectan	112	https://pdsimage2.wr.usgs.gov/archi

	EN0242671923M	gular	m/pixel	ve/mess-e_v_h-mdis-2-edr-rawdata-v1.0/
**Fig. 3c**	MDIS NAC EN0211981012M, EN0211981014M	Equirectangular	67 m/pixel	https://pdsimage2.wr.usgs.gov/archive/mess-e_v_h-mdis-2-edr-rawdata-v1.0/
**Fig. 3d**	MDIS NAC EN0242713760M	Equirectangular	54 m/pixel	https://pdsimage2.wr.usgs.gov/archive/mess-e_v_h-mdis-2-edr-rawdata-v1.0/
**Fig. 8a,b**	Vesta Dawn FC HAMO Global DTM	Polar Stereographic, scale factor=1.65	93 m/pixel	https://astrogeology.usgs.gov/search/map/Vesta/Dawn/DLR/HAMO/Vesta_Dawn_HAMO_DTM_DLR_Global_48ppd
**Fig. 8c,d, S Fig. 6**	Vesta Dawn FC HAMO Global Shaded Relief	Polar Stereographic, scale factor=1.65	93 m/pixel	https://astrogeology.usgs.gov/search/map/Vesta/Dawn/DLR/HAMO/Vesta_Dawn_HAMO_ClrShade_DLR_Global_48ppd
**Fig. 9c**	Selene Kaguya Terrain Camera TCO_MAPm04_N45E306N42E309SC	Mercator	7.4 m/pixel	https://darts.isas.jaxa.jp/planet/pdap/selene/index.html.en
**Fig. 10a,b, S Fig. 7b,c**	Mars Odyssey THEMIS-IR Day Global Mosaic	simple cylindrical	100 m/pixel	https://pds-imaging.jpl.nasa.gov/volumes/ody.html
**Fig. 10c**	MRO CTX F05_037578_1968_XN_16N211W	Equirectangular	5 m/pixel	https://pds-imaging.jpl.nasa.gov/portal/mro_mission.html
**S Fig. 1**	Mariner 10 global mosaic	Orthographic, centered at 100°W, 3°N	1 km/pixel	http://ser.sese.asu.edu/M10/IMAGE_ARCHIVE/MOSAICS/index.html
**S Fig. 5b**	MDIS NAC EN0243883525M, EN0243883532M, EN0243912333M, EN0243912340M	Equirectangular	19 m/pixel	https://pdsimage2.wr.usgs.gov/archive/mess-e_v_h-mdis-2-edr-rawdata-v1.0/
**S Fig. 7a**	Crater density map of Mars	Robinson	0.05°/pixel	https://hive.curtin.edu.au/research/CDA-94M-release/
**S Fig. 8**	Voyager-Galileo SSI Global Mosaic	Equirectangular	500 m/pixel	https://astrogeology.usgs.gov/search/map/Europa/Voyager-Galileo/Europa_Voyager_GalileoSSI_global_mosaic_500m

Supplementary References

1. Dobrovolskis, A. Ejecta patterns diagnostic of planetary rotation. *Icarus* **47**, 203–219 (1981).

2. Otto, K. A. et al. The Coriolis effect on mass wasting during the Rheasilvia impact on asteroid Vesta. *Geophys. Res. Lett.* **43(24)**, 12–340 (2016).

3. Lagain, A. et al. The Tharsis mantle source of depleted shergottites revealed by 90 million impact craters. *Nat. Commun.* **12(1)**, 1–9 (2021).

4. Carr, M. H. et al. Evidence for a subsurface ocean on Europa. *Nature* **391(6665)**, 363–365 (1998).

5. Bierhaus, E. B., Chapman, C. R. & Merline, W. J. Secondary craters on Europa and implications for cratered surfaces. *Nature* **437(7062)**, 1125–1127 (2005).
